# Rapid whole-brain quantitative MT imaging

**DOI:** 10.1016/j.zemedi.2023.02.005

**Published:** 2023-04-03

**Authors:** Roya Afshari, Francesco Santini, Rahel Heule, Craig H. Meyer, Josef Pfeuffer, Oliver Bieri

**Affiliations:** a Division of Radiological Physics, Department of Radiology, University Hospital Basel, Basel, Switzerland; b Department of Biomedical Engineering, University of Basel, Basel, Switzerland; c BAMM group, Department of Biomedical Engineering, University of Basel, Basel, Switzerland; d High Field Magnetic Resonance, Max Planck Institute for Biological Cybernetics, Tübingen, Germany; e Department of Biomedical Magnetic Resonance, University of Tübingen, Tübingen, Germany; f Department of Biomedical Engineering, University of Virginia, Charlottesville, VA, USA; g Siemens Healthcare, Application Development, Erlangen, Germany

**Keywords:** Magnetization transfer, qMT, Spiral, B_1_, Correction, Brain

## Abstract

**Purpose:**

To provide a robust whole-brain quantitative magnetization transfer (MT) imaging method that is not limited by long acquisition times.

**Methods:**

Two variants of a spiral 2D interleaved multi-slice spoiled gradient echo (SPGR) sequence are used for rapid quantitative MT imaging of the brain at 3 T. A dual flip angle, steady-state prepared, double-contrast method is used for combined B_1_ and-T_1_ mapping in combination with a single-contrast MT-prepared acquisition over a range of different saturation flip angles (50 deg to 850 deg) and offset frequencies (1 kHz and 10 kHz). Five sets (containing minimum 6 to maximum 18 scans) with different MT-weightings were acquired. In addition, main magnetic field inhomogeneities (ΔB_0_) were measured from two Cartesian low-resolution 2D SPGR scans with different echo times. Quantitative MT model parameters were derived from all sets using a two-pool continuous-wave model analysis, yielding the pool-size ratio, F, their exchange rate, k_f_, and their transverse relaxation time, T_2r_.

**Results:**

Whole-brain quantitative MT imaging was feasible for all sets with total acquisition times ranging from 7:15 min down to 3:15 min. For accurate modeling, B_1_-correction was essential for all investigated sets, whereas ΔB_0_-correction showed limited bias for the observed maximum off-resonances at 3 T.

**Conclusion:**

The combination of rapid B_1_-T_1_ mapping and MT-weighted imaging using a 2D multi-slice spiral SPGR research sequence offers excellent prospects for rapid whole-brain quantitative MT imaging in the clinical setting.

## Introduction

In its simplest and traditional form, magnetization transfer (MT) contrast [1] is quantified from the acquisition of two scans: one without and one with the MT-preparation module [2]. The two signals are then condensed within the so-called magnetization transfer ratio (MTR) and a great effort has been undertaken to ensure high reproducibility taking into account intrinsic, as well as possible extrinsic, confounding factors [3]. It has, however, also been realized that the phenomenological reduction of a complex tissue system down to a single parameter may lack pathologic specificity making MTR results incomplete and controversial, especially in the brain [4]. As a result, biophysical models of MT have been developed that allow the quantitative estimation of the compartmental tissue properties. To this end, two-compartment (or binary) spin-bath models are most commonly considered to yield quantitative MT parameter (qMT) estimates, such as the pool-size ratio of the two compartments, their rate of magnetization exchange, and the compartmental relaxation properties [5], [6], [7], [8]. As with any other quantitative MRI method, however, accurate estimation of the underlying tissue model parameters may depend on deviations from the presumed radiofrequency (RF) excitation field (B_1_), and inhomogeneities in the main magnetic field (ΔB_0_).

Generally, qMT imaging requires multiple MT-weighted measurements and thus prolonged image acquisitions which may prevent widespread clinical use and applicability [6], [8]. This can, for instance, be addressed by a reduction of the number of free MT model parameters (thus reducing the number of measurements) [8], [9].Alternatively, rapid imaging concepts, such as MT-sensitized balanced steady state free precession (bSSFP) [10] or highly efficient k-space sampling schemes, such as MT-sensitized single-shot echo planar imaging (EPI) [11] can be used to reduce the scan time for qMT imaging down to ∼10-15 min.

Only recently, a spiral imaging concept was proposed for rapid whole-brain MTR imaging with intrinsic B_1_-correction within less than one minute [12]. The method takes advantage of an MT-weighted multi-slice spiral spoiled gradient echo (SPGR) research sequence offering whole-brain coverage for the acquisition of a single MT-weighted volume within 20 s. Notably, the same underlying spiral SPGR research sequence was also suggested for rapid intrinsically B_1_-corrected whole-brain T_1_ mapping in less than one minute [13], [14], [15]. In this work, we thus propose to fuse these two concepts for rapid, whole-brain, in-vivo qMT imaging using a two-pool model analysis. We will show that the proposed method allows rapid whole-brain qMT imaging in less than typically 5 minutes thus being compatible with the clinical workflow.

## Methods

### Imaging sequences and image reconstruction

MT-weighted imaging was performed with an interleaved, multi-slice, spiral SPGR research sequence, as described in [12], [13]. The MT-preparation module had a duration of 19.1 ms and consisted of a 17.92 ms non-selective, unapodized, Gaussian-shaped, radio-frequency saturation pulse with variable frequency offset (Δ) and variable flip angle (β) and crusher gradients. Slice selection was performed with a sinc-shaped RF pulse of 0.6 ms duration and a time-bandwidth-product of 1.6. A flip angle of α = 25 deg and a slice thickness of 3 mm was used. The total acquisition duration of the imaging kernel (including slice selection, spiral readout, and crusher gradients) was 9.75 ms.

**Figure 1:**
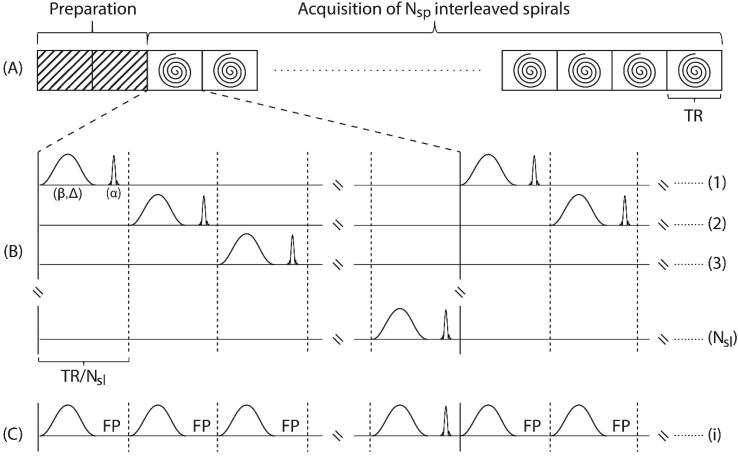
Scheme of the proposed MT-weighted multi-slice interleaved spiral SPGR sequence. (A) After a dummy preparation period of two TR, data in each slice is sampled by N_sp_ spiral interleaves separated by TR. (B) Within each TR, N_sl_ slices are acquired in an interleaved manner. For each slice, MT preparation with a non-selective Gaussian-shaped saturation pulse of variable frequency offset (Δ) and variable flip angle (β) preceded slice excitation with a flip angle (α) using a sinc-shaped excitation pulse. (C) From the interleaved slice acquisition, each slice excitation effectively preceded a train of N_sl_ MT-saturation pulses separated by short free precession (FP) periods.

For each slice, *N_sp_* = 20 spiral interleaves in combination with an acceleration factor of two were used; thus effectively reducing the acquisition to 10 spiral readouts per slice. Data was reconstructed online on the scanner with an in-plane resolution of 1.3×1.3 mm^2^ using a spiral version of the “iTerative Self-consistent Parallel Imaging Reconstruction” method (SPIRiT) [16]. An auto-stop criterion was used, also when the k-space was fully sampled at the Nyquist rate, to implicitly derive the optimal density compensation function for the gridding algorithm. A single high-resolution MT-weighted volume was reconstructed from the acquisition of *N_sl_* = 50 interleaved slices with a repetition time (TR) of 1650 ms (Fig. 1). The overall acquisition time for a single MT-weighted whole-brain volume was 19.8 s; including a dummy preparation period of 2×TR (i.e., without readout) to reach the steady state for tissues (see Fig. 1A).

In addition, a T_1_-map and a low-resolution B_1_-map were acquired based on the same spiral research sequence, described in detail in [13]. Generally, B_1_ field inhomogeneities lead to local deviations from the nominal flip angle, $\alpha_{\mathrm{nom}}\to \alpha_{\mathrm{act}}=\zeta_{B1}∙\alpha_{\mathrm{nom}}$, using a scaling factor $\zeta_{B1}$. Essentially, the same resolution and number of slices were used as for the MT-weighted scans [17]. Two scans with optimal variable flip angles (VFA) of 17 deg and 80 deg for a TR of 250 ms were used. The overall acquisition time for the combined B_1_ ($\equiv \zeta_{B1}$) and-T_1_ mapping was 53 s.

Moreover, a ΔB_0_ map (as usual given in Hz via the association $\Delta B_{0}\mapsto \gamma ∙\Delta B_{0}$) was derived from the acquisition of two multi-slice GRE volumes with different echo times, TE_1_ = 1.45 ms and TE_2_ = 1.90 ms. Each acquisition had 50 slices with slice thickness of 3 mm measured with a TR of 180 ms, in-plane resolution of 1.3×1.3 mm^2^, and FOV of 256×256 mm^2^. The total acquisition time for the ΔB_0_ mapping was 22 s.

### MT signal modeling and numerical simulation

From the interleaved slice acquisition (see Fig. 1B), pulsed MT-contrast in each slice is generated by a train of *N*_sl_ MT pulses separated by short free precession periods (see Fig. 1C), having an effective duration of 1650 ms (*TR*) and corresponding mean saturation rate $\overset{¯}{W}$ [18]:

(1) $$\overset{¯}{W}=N_{\mathrm{sl}}\frac{\pi }{\mathrm{TR}}\int_{0}^{T_{\mathrm{RF}}}\omega_{1}^{2}\left(t\right)dtG\left(\Delta \right)$$

where $G\left(\Delta \right)$ is the absorption line shape, which is assumed to be super-Lorentzian for tissues, and $\overset{¯}{W}$ depends on the duration $T_{\mathrm{RF}}$ and the shape $\omega_{1}\left(t\right)=\gamma \left|B_{1}\left(t\right)\right|$ of the MT pulse.

Consequently, if a fractional saturation of the free pool due to the given train of MT pulses is avoided, the steady state signal S may approximate the situation that is established by a long period of continuous-wave irradiation of the restricted pool protons (see Eq. A5 in ref. [5]):

(2) $$S=c.M_{0,f}\frac{R_{1,r}k_{f}+R_{1,r}R_{1,f}+R_{1,f}k_{r}+\overset{¯}{W}R_{1,f}}{R_{1,r}R_{1,f}+R_{1,r}k_{f}+R_{1,f}k_{r}+\overset{¯}{W}R_{1,f}+\overset{¯}{W}k_{f}}$$

where $M_{0,f}$ is the equilibrium magnetization of the free pool; $R_{1,r}$ and $R_{1,f}$ are the longitudinal relaxation rates of the restricted and of the free pool, respectively; $k_{f}$ and $k_{r}$ are the first order exchange rates between the free and the bound pool protons, respectively; and c collects all other parameters, such as coil sensitivities. The exact value of R_1r_ has only a minor influence on MT imaging [5]. Following Yarnykh [8], $R_{1,r}=R_{1,obs}$ is chosen, leading to $R_{1,f}=R_{1,obs}$ (cf. Eq. [10] in Ref. [5]). In this work, $R_{1,obs}=R_{1,r}=R_{1,f}=1/T_{1}$ is derived from a spiral VFA measurement. Overall, excellent agreement between the spiral VFA method and IR reference measurements were observed [15].

For validation purposes, numerical simulations of the set of coupled Bloch equations including the exchange of longitudinal magnetization in the two-pool model were performed as described in detail, elsewhere [19]. Within the context of this work, however, perfect spoiling of transverse magnetization was assumed (note that an interleaved acquisition scheme with a TR of 1650 ms was used) and it was presumed that the offset frequency of the MT-saturation pulse will be chosen large enough to avoid any direct fractional saturation effects of the free pool. For numerical simulation of the steady state signal, we thus proceeded as follows:

MT-pulses were simulated using the coupled Bloch equations, which are reduced to a set of two coupled differential equations for the longitudinal magnetization components,

(3a) $$\frac{dM_{z,f}}{\mathrm{dt}}=R_{1,f}\left(M_{0,f}-M_{z,f}\right)-k_{f}M_{z,f}+k_{r}M_{z,r}$$

(3b) $$\frac{dM_{z,r}}{\mathrm{dt}}=R_{1,r}\left(M_{0,r}-M_{z,r}\right)+k_{f}M_{z,f}+k_{r}M_{z,r}-\pi \omega_{1}^{2}\left(t\right)G\left(\Delta \right)$$

Note that the train of MT pulses is interleaved by free-precession periods which were simulated by setting $\omega_{1}$ in Eq. (3b) equal to zero. At the end of the pulsed-MT-free-precession train, RF excitation of the free pool occurs, which was assumed to act instantaneously on the longitudinal component of the free pool. The overall succession of MT-pulses, free precession periods, and RF excitation, was repeated until a steady state was reached; which was typically established after two to three repetitions.

A standard ODE solver was used to simulate the time evolution of the longitudinal magnetization components according to Eq. 3 with common white and gray matter MT tissue parameters [20].

### Data evaluation

Whole-brain voxel-wise B_1_ maps, together with B_1_-corrected $T_{1,B1}$ and B_1_-uncorrected $T_{1}$ maps were generated from the two VFA spiral SPGR scans as described elsewhere [13], whereas ΔB_0_ maps were derived from the phase factor $e^{-i\gamma {\Delta B}_{0}\left(TE_{2}-TE_{1}\right)}$, relating to the two low-resolution SPGR phase images, acquired with different echo times TE_1_ and TE_2_. Generally, B_1_ effects enter the MT model (cf. Eqs. (1), 2) by: (*i*) a modulation of $\omega_{1}^{2}\mapsto \zeta_{B1}^{2}∙\omega_{1}^{2}$, and (*ii*) a correction of the observed $T_{1}\to T_{1,B1}$; field inhomogeneities lead to a shift of the off-resonance irradiation frequency $\Delta \mapsto \Delta -\Delta B_{0}$. Finally, voxel-wise estimates for the pool-size ratio F, k_f_, and T_2r_ were derived from a non-linear least-squares (NLLS) fit of Eqs. (1) and (2) to a set *S* of MT-weighted signal observations.

The standard software package FSL (FMRIB Software Library v6.0, Oxford, UK) was used for co-registration and skull stripping of the MRI datasets. All other image postprocessing, simulations and visualizations were performed using MATLAB R2019a (The MathWorks, Natick, MA).

### In-vivo imaging

Three volunteers were scanned at 3 T (MAGNETOM Prisma, Siemens Healthcare, Erlangen, Germany) using a 20-channel receive head coil. Written informed consent was obtained from participants and measurements were approved by our local ethics committee.

Quite some effort has been undertaken to find optimal sets of MT sampling points that yield robust MT parameter estimates within clinically acceptable scan times [17], [21]. For 3D scans, about 10 measurements are required [17], [21]. Similarly, Ramani et al. [6] observed a minimum number of about 10 MT measurements using a 2D multi-slice approach with six offset frequencies (Δ) ranging from 1 – 15 kHz using three different saturation flip angles (β). Furthermore, it was observed that at least one point (potentially better two points) with either large Δ or small β (and thus with no MT weighting) should be included [21].

Following Ramani [6], a lower limit of Δ_min_ = 1 kHz was used to mitigate direct saturation effects (cf. Eq. (2)), whereas the upper bound for the saturation flip angle was β_max_ = 850 deg due to limits from the specific absorption rate (SAR). The upper limit of Δ_max_ = 10 kHz was configured to maximize the MT signal sensitivity to F and T_2r_ [22]. No MT weighting was achieved from using a lower bound of β_min_ = 50 deg. In order to explore a range of 18 down to 6 MT sampling points for subsequent qMT parameter estimation, two base sets of MT-weighted data were acquired:

$$S_{1}:=\left\{\Delta \;=1,\;10\;\left[kHz\right]\wedge \;\beta =50,\;150,\;250,\;350,\;450,\;550,\;650,\;750,\;850\;\left[deg\right]\right\}$$

$$S_{2}:=\left\{\Delta \;=1,\;10\;\left[kHz\right]\wedge \;\beta =50,\;184,\;316,\;450,\;584,\;716,\;850\;\left[deg\right]\right\}$$

From the base sets, the following subsets were synthesized and also analyzed:

$$S_{3}:=\left\{\Delta \;=1,\;10\;\left[kHz\right]\wedge \;\beta =50,\;250,\;450,\;650,\;850\;\left[deg\right]\right\}$$

$$S_{4}:=\left\{\Delta \;=1,\;10\;\left[kHz\right]\wedge \;\beta =50,\;316,\;584,\;850\;\left[deg\right]\right\}$$

$$S_{5}:=\left\{\Delta \;=1,\;10\;\left[kHz\right]\wedge \;\beta =50,\;450,\;850\;\left[deg\right]\right\}$$

The scan times for the base sets S_1_ (2 × 9 scans) and S_2_ (2 × 7 scans) were 6:00 min and 4:40 min; respectively. The synthetic data sets S_3_ (2 × 5 scans), S_4_ (2 × 4 scans) and S_5_ (2 × 3 scans) have notional scan times of 3:20 min, 2:40 min and 2:00 min, respectively. For qMT imaging, this leads to scan times that range from 7:15 min down to 3:15 min (including 53 s for the two VFA scans for B_1_-T_1_-mapping and 22 s for the two GRE scans for ΔB_0_ mapping).

## Results

**Figure 2:**
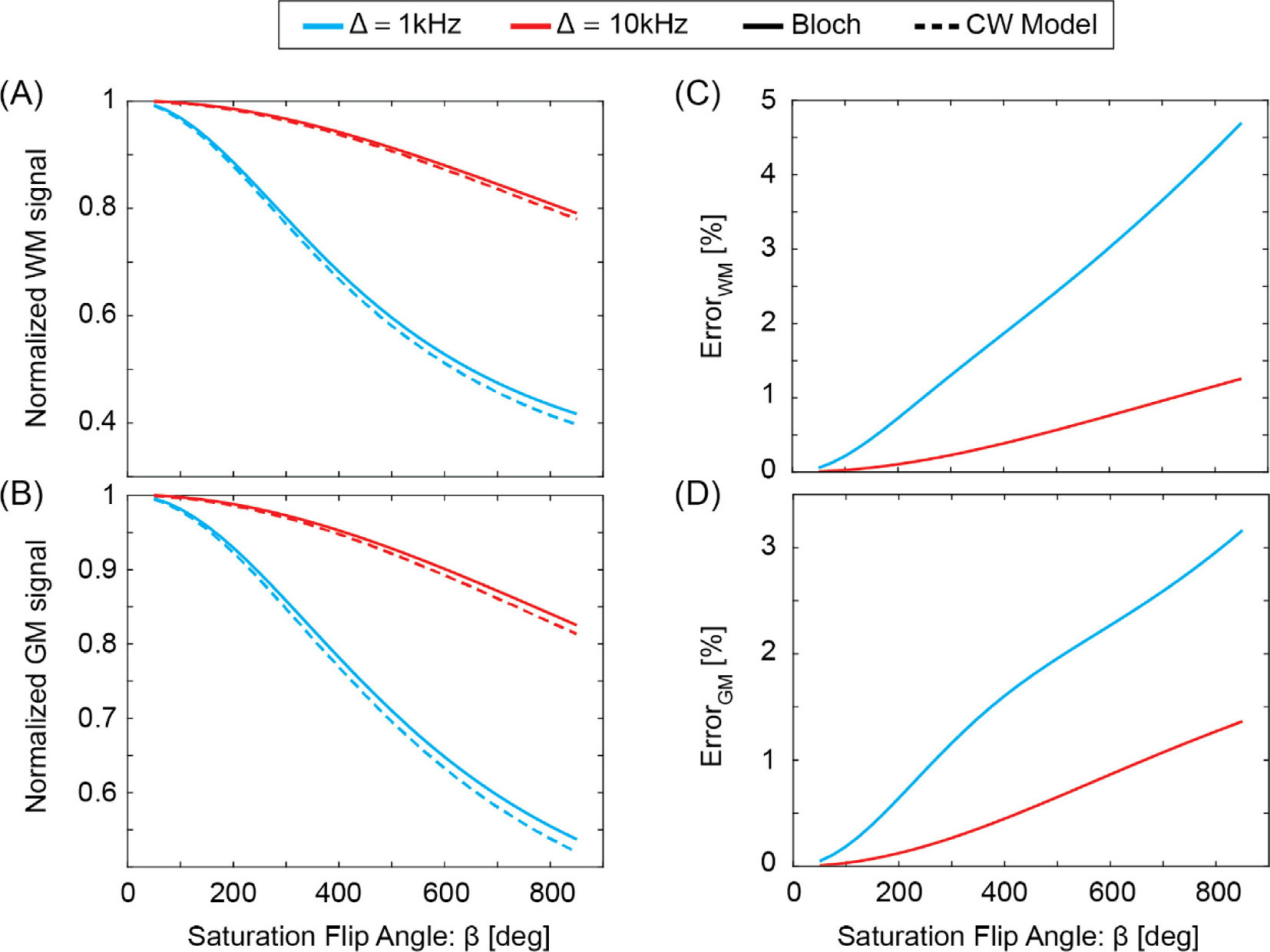
Bloch simulation (solid line) versus CW two-pool model predictions (Eq. (2), dashed line) for typical MT parameter values at 3 T for (A) white matter (F = 13.7 %, k_f_ = 4.3 s^−1^, R_1,f_ = 1.17 s^−1^, T_2r_ = 12 µs), (B) gray matter (F = 6.2 %, k_f_ = 1.8 s^−1^, R_1,f_ = 0.8 s^−1^, T_2r_ = 10 µs), and their relative difference (C, D) as a function of the saturation flip angle β at two different offset irradiation frequencies.

Fig. 2 shows a comparison of the continuous-wave (CW) approximation (Eq. (2)) with numerical simulations for white and gray matter using parameter values from [20]. Within the range of experimentally applied offset irradiation frequencies (1 and 10 kHz) and saturation flip angles (β = 50 to 850 deg) the CW solution overestimates MT-saturation effects by maximal 1.5% at 10 kHz, and at 1 kHz the maximum relative error amounts to less than 5% for WM and less than 3.5% for GM. In summary, good agreement between the CW solution and the numerical simulations was found.

**Figure 3:**
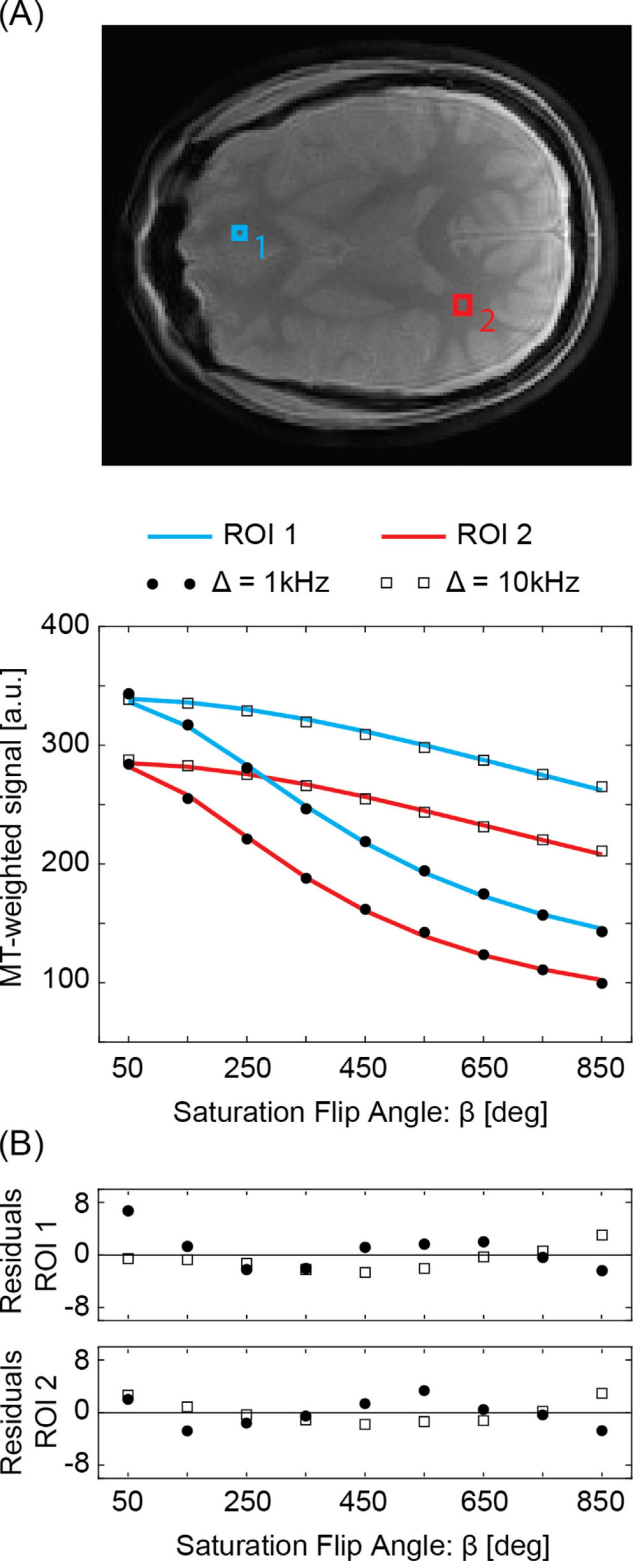
(A) In-vivo CW two-pool model analysis (solid line) of MT-weighted signals from set S_1_ for two regions of interest, located in cortical brain tissue (ROI1, blue box, ΔB_0_ = 2.9 Hz, $\zeta_{B1}$ =1.11) and in the white matter (ROI2, red box, ΔB_0_ = 90 Hz, $\zeta_{B1}$ = 0.98). Square dots correspond to regional average signals from the MT-weighted images acquired with an offset frequency of Δ = 10 kHz, whereas round dots are representing regional average signals from MT-weighted images acquired with Δ = 1 kHz. (B) Fitting residuals.

Fig. 3 summarizes in-vivo CW model fitting results (Eqs. (1), (2)) for the average signal of two small, presumably homogeneous, regions of interest (ROI1 = 42 pixels, ROI2 = 24 pixels) using data from the 18-samples set S_1_. Overall, the fitting residuals indicate appropriate modelling of the data. Upon B_1_ and ΔB_0_ correction, T_1_ and qMT parameter estimates for ROI1 (ΔB_0_ = 2.9 Hz, $\zeta_{B1}$= 1.11) change for T_1_ from 1182 ms to 873 ms, for F from 14.3 ± 1.2 % to 15.0 ± 1.2 %, for k_f_ from 3.1 ± 0.4 s^−1^ to 4.2 ± 0.6 s^−1^, and for T_2r_ from 12.0 ± 0.4 μs to 12.0 ± 0.4 μs. For ROI2 (ΔB_0_ = 90 Hz, $\zeta_{B1}$ = 0.98), T_1_ is changed from 1526 ms to 1597 ms, F is changed from 7.7 ± 0.7 % to 7.4 ± 0.7 %, k_f_ is changed from 1.9 ± 0.3 s^−1^ to 1.8 ± 0.3 s^−1^, and T_2r_ is changed from 11.3 ± 0.5 μs to 11.2 ± 0.5 μs. In summary, k_f_ is most sensitive to and severely affected only by B_1_ field miscalibrations, F is affected by both B_1_ and ΔB_0_ field variations but more strongly by B_1_ than ΔB_0_ field miscalibrations, whereas the overall bias in T_2r_ appears neglectable.

**Figure 4:**
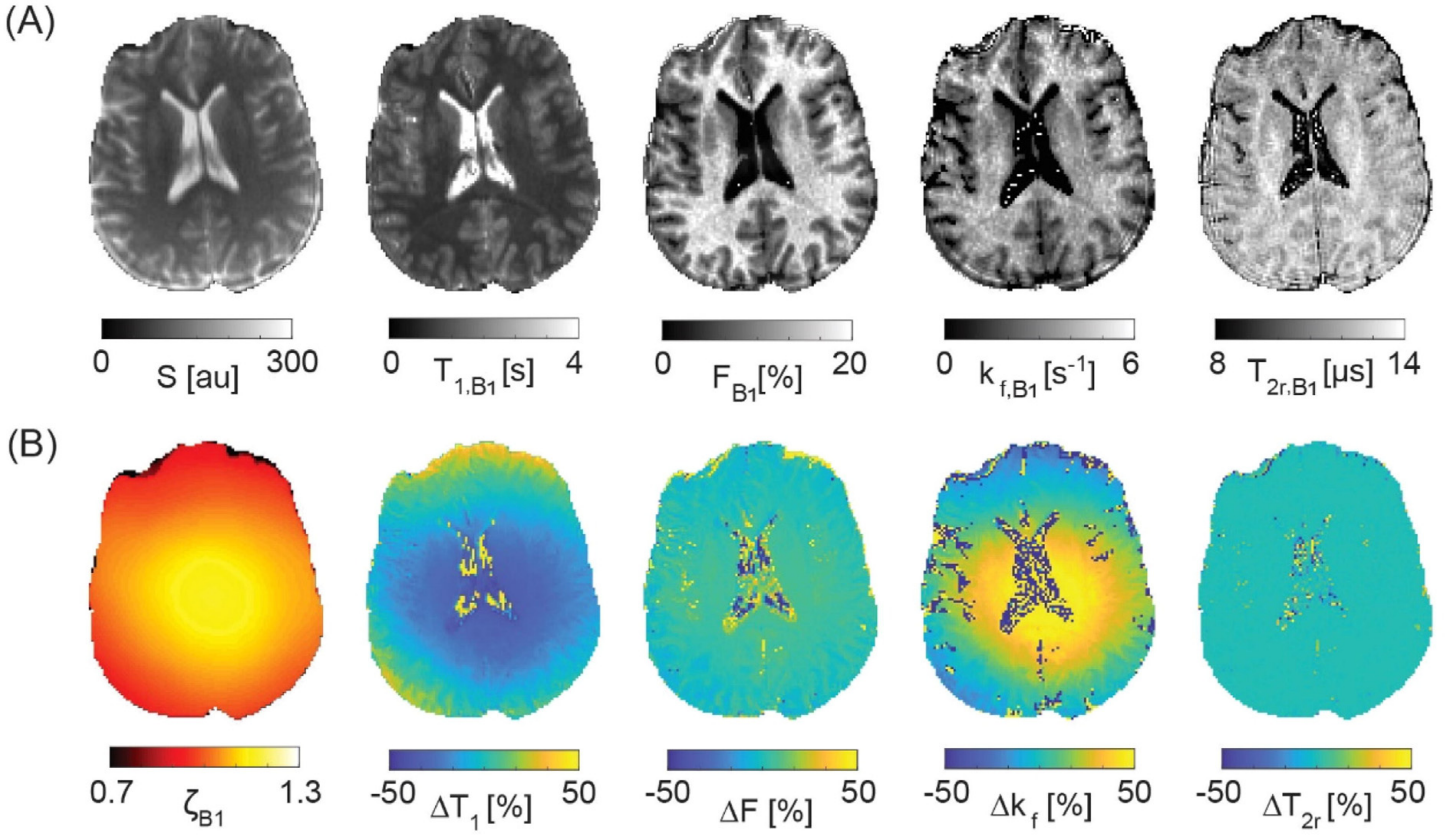
B_1_ bias on qMT model parameters. (A) B_1_-corrected T_1,B1_, F_B1_, k_f,B1_, and T_2r,B1_ parameter maps. (B) B_1_-map together with its relative contribution to uncorrected T_1_, F, k_f_, and T_2r_ parameter estimates.

**Figure 5:**
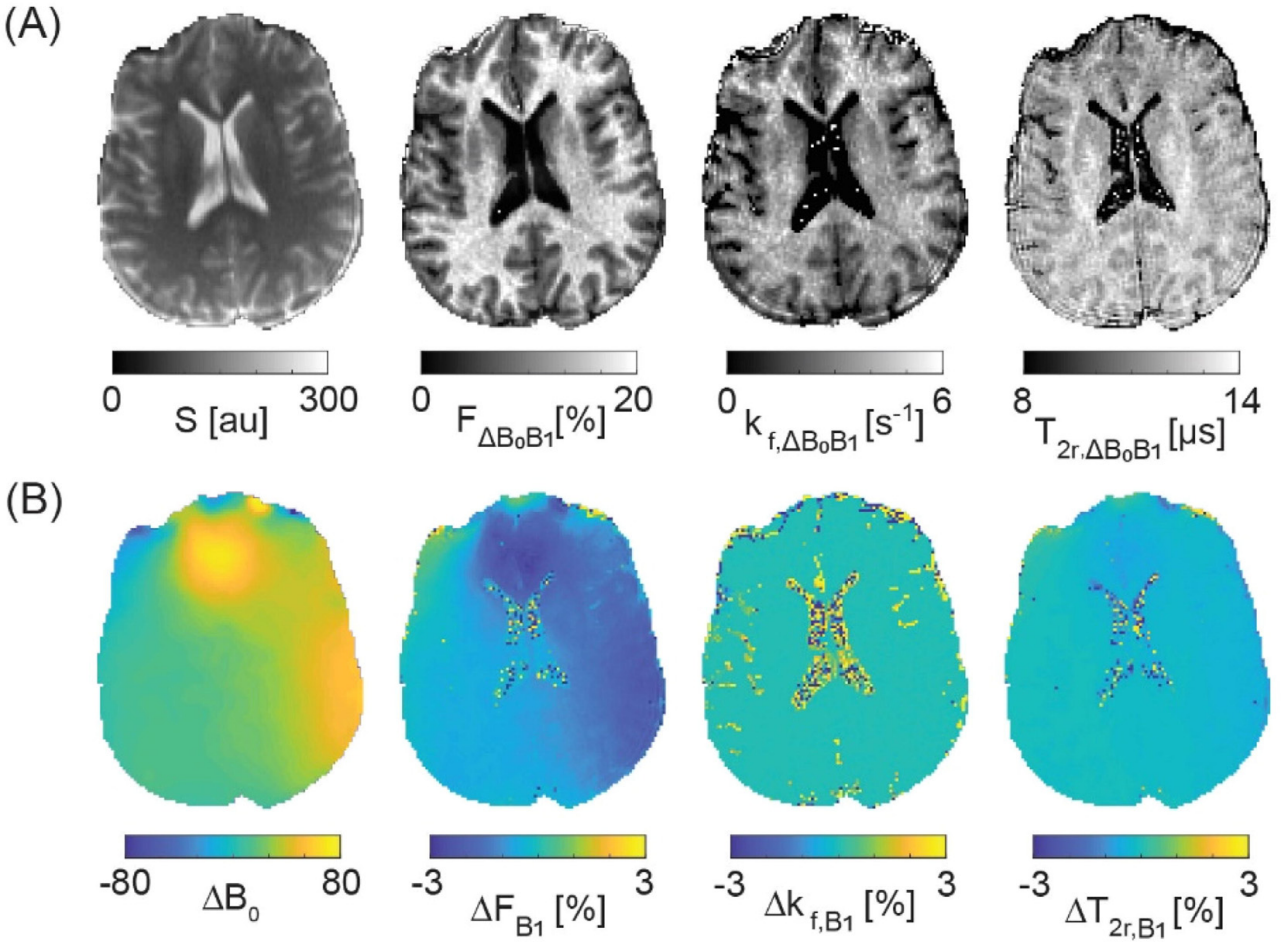
ΔB_0_ bias on B_1_-corrected qMT model parameters. (A) ΔB_0_-B_1_-corrected F_ΔB0B1_, k_f,__ΔB0B1_ and T_2r,__ΔB0B1_ parameter maps. (B) ΔB_0_-map together with its relative contribution to B_1_-corrected F_B1_, k_f,B1_ and T_2r,B1_ parameter estimates.

This finding is further corroborated and summarized in Fig. 4, showing the overall bias as introduced by B_1_ field miscalibrations only on both T_1_ and qMT parameter maps for a single axial slice using again the data from the 18-samples set S_1_. The typical B_1_ range of about ±25 % at 3 T results in an about two-fold stronger bias in k_f_ (±50 %), whereas variations in F are about three-fold less (±8 %). Generally, T_2r_ estimates are not affected by B_1_. This is in contrast to ΔB_0_ field variations where the maximum observed local off-resonances near the nasal cavity in the order of about +100 Hz lead to local variations in F by a few percent (less than about -3 %) and to overall changes in T_2r_ by less than about 1 %. The forward exchange rate, k_f_, is unaffected (see Fig. 5 for the assessment of ΔB_0_-field miscalibrations on qMT parameter maps).

**Figure 6:**
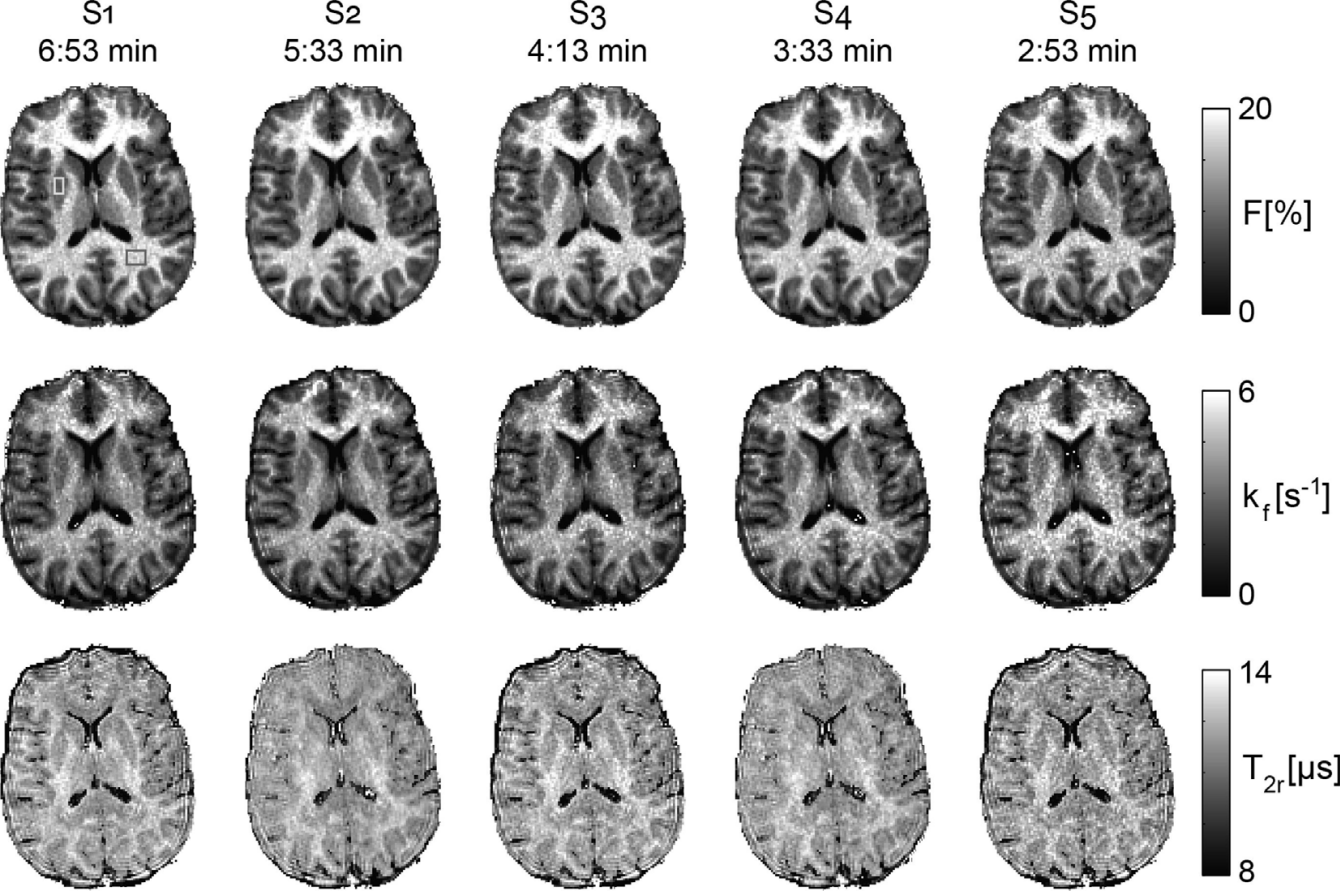
Effect of sampling point reduction on B_1_-corrected qMT parameter estimates.

Figure 3, Figure 4, Figure 5 were derived using the 18-samples set S_1_. In Fig. 6, the effect of reduced sample sizes (and thus shortened scan times) on qMT parameter estimation is shown. Generally, qMT parameter maps show no marked fitting failures, even for the 6-samples set S_5_. As can be expected, however, the uncertainty in the parameter estimates increases with decreasing number of samples: for a ROI in WM (cf. Fig. 6), F and k_f_ estimates change from 16.3 ± 1.1 % in set S_1_ to 16.2 ± 2.1 % in the 10-samples set S_3_ to 15.2 ± 5.2 % for the 6-samples set S_5_, and from 3.7 ± 0.4 s^−1^ in S_1_ to 3.8 ± 0.8 s^−1^ in S_3_ to 4.2 ± 2.6 s^−1^ in S_5_, respectively. Overall, a similar trend is observed for T_2r_ and similar observations were made for gray matter.

## Discussion

Generally, qMT parameter mapping requires a set of measurements with different MT-weighting and might thus suffer from over-lengthy scan time requirements in the clinical setting. One obvious strategy to reduce scan time is to reduce the number of MT sampling points and state-of-the-art methods typically require about a minimum of 10 sampling points [17], [21]. Alternatively, efficient sequences that offer short repetition times, such as bSSFP, can be used to reduce the overall scan time [10]. Ideally, however, efficient signal acquisition is combined with a low number of sampling points. To this end, in this work, rapid whole-brain qMT imaging was explored using an interleaved multi-slice spiral research sequence at 3 T for combined B_1_ and T_1_ mapping, as well as for the acquisition of a set of MT-weighted signals with different saturation powers and off-resonances. Due to the interleaved multi-slice acquisition, a simple two-compartment CW MT model could be used to model the data. Without ΔB_0_ correction, the total scan time of the investigated B_1_-corrected qMT protocols ranges from 6:53 min for the 18-samples set down to 4:13 min for the 10-samples set and finally down to 2:53 min for the 6-samples set, yielding 50 slices with a resolution of 1.3×1.3×3.0 mm^3^ for T_1_, F, k_f_ and T_2r_.

Conventional qMT methods use sampling schemes covering a broad range of off-resonances (Δ) at a rather limited number of MT saturation powers (β) [6], [19], [23]. In this work, MT contrast was explored using a broad range of saturation powers measured at two off-resonances. The latter approach was preferred since conventional methods include low off-resonances (Δ < 1kHz) but Eq. (2) does not account for direct saturation effects. Thus, MT saturation cannot be explored at low off-resonance frequencies. No such restriction, however, applies for the MT saturation power. Moreover, a different signal model is used, and it is a priori unclear whether conventional optimal sampling schemes apply here as well.

Overall, no systematic investigation was performed to find the optimal {β,Δ} sampling pattern for a given number of measurements. Thus, different {β,Δ} patterns might be found that even lead to more robust parameter estimates. The 6-point sampling pattern, however, embraces 3 MT saturation powers at two offset frequencies and comes close to the minimal number of scans required [17], indicating a reasonable choice of the sampling pattern. The 10-point sampling scheme together with the combined B_1_-T_1_ mapping can be performed in less than 5 minutes. Overall, estimated MT parameters were well within the range of what was previously reported [19].

In contrast to related work [24], a higher B_1_ bias is observed for F upon using B_1_ uncorrected VFA T_1_ values (for the settings used here, about three-fold). Consequently, appropriate B_1_ correction appears mandatory but requires no extra scan time using the proposed rapid dual-contrast VFA approach [13]. This is in contrast to ΔB_0_, where the typical maximum bias on F is limited to a few percent and is negligible for all other qMT parameter estimates. Thus, the bias in F from ΔB_0_ is on the order of the uncertainty of the measurement, especially for measurements using 10 or less sampling points. Thus, whether ΔB_0_ correction needs to be performed depends on the desired accuracy for the qMT parameter estimates, as well as whether the additional required 22 s needs to be spent or not.

## Conclusion

A fast qMT imaging method is proposed based on two variants of an interleaved multi-slice spiral research sequence at 3 T. B_1_-correction was mandatory for appropriate MT parameter estimation while the overall effect of ΔB_0_ can be neglected. The 10-point MT-weighted sampling scheme together with the B_1_-T_1_ acquisition offers whole-brain qMT imaging with clinically relevant resolutions in less than 5 minutes and thus offers excellent prospects for widespread clinical translation and use.

## Declaration of Competing Interest

The authors declare the following financial interests/personal relationships which may be considered as potential competing interests: One of the authors, Josef Pfeuffer, is employed by Siemens Healthineers.
